# Neurofeedback in patients with frontal brain lesions: A randomized, controlled double-blind trial

**DOI:** 10.3389/fnhum.2022.979723

**Published:** 2022-09-15

**Authors:** Christine Annaheim, Kerstin Hug, Caroline Stumm, Maya Messerli, Yves Simon, Margret Hund-Georgiadis

**Affiliations:** REHAB Basel, Klinik für Neurorehabilitation und Paraplegiologie, Basel, Switzerland

**Keywords:** neurorehabilitation, neurofeedback (NFB), brain recovery, frontal brain injury, cognitive dysfunction, brain computer interface, infra-low frequency neurofeedback

## Abstract

**Background:**

Frontal brain dysfunction is a major challenge in neurorehabilitation. Neurofeedback (NF), as an EEG-based brain training method, is currently applied in a wide spectrum of mental health conditions, including traumatic brain injury.

**Objective:**

This study aimed to explore the capacity of Infra-Low Frequency Neurofeedback (ILF-NF) to promote the recovery of brain function in patients with frontal brain injury.

**Materials and methods:**

Twenty patients hospitalized at a neurorehabilitation clinic in Switzerland with recently acquired, frontal and optionally other brain lesions were randomized to either receive NF or sham-NF. Cognitive improvement was assessed using the Frontal Assessment Battery (FAB) and the Test of Attentional Performance (TAP) tasks regarding intrinsic alertness, phasic alertness and impulse control.

**Results:**

With respect to cognitive improvements, there was no significant difference between the two groups after 20 sessions of either NF or sham-NF. However, in a subgroup of patients with predominantly frontal brain lesions, the improvements measured by the FAB and intrinsic alertness were significantly higher in the NF-group.

**Conclusion:**

This is the first double-blind controlled study using NF in recovery from brain injury, and thus also the first such study of ILF NF. Although the result of the subgroup has limited significance because of the small number of participants, it accentuates the trend seen in the whole group regarding the FAB and intrinsic alertness (*p* = 0.068, *p* = 0.079, respectively). We therefore conclude that NF could be a promising candidate promoting the recoveryfrom frontal brain lesions. Further studies with larger numbers of patients and less lesion heterogeneity are needed to verify the usefulness of NF in the neurorehabilitation of patients with frontal brain injury (NCT02957695 ClinicalTrials.gov).

## Introduction

### Frontal brain dysfunction

Patients with frontal brain lesions suffer from a variety of symptoms including apathy, attention deficits, impaired executive functions, lack of impulse control, and impaired emotional regulation. One typical feature that complicates these problems is the so-called anosognosia, namely patient’s lack of awareness of the deficits (Malloy and Grace, 2005; Prigatano, 2005; Arnould et al., 2016). The resulting behavioral and social problems often are a heavy burden for family members or for institutions (Wells et al., 2005; Guevara et al., 2016). At the same time, these symptoms are a major challenge to treat since conventional therapies, such as occupational therapy or neuropsychological training, are commonly not sufficient to overcome the problems of self-regulation.

### Neurofeedback

Neurofeedback (NF) is a psychophysiological procedure where cerebral regulation is promoted through real-time feedback to a person based on his or her own brain activity as measured by electroencephalography (EEG). The patient is involved in a feedback loop whereby brain activity is recorded and instantaneously translated into visual, auditory and tactile signals that can be perceived by the individual. With respect to mechanisms of action, functional MRI (fMRI)-based NF studies have shown that basic principles of neuroplasticity are involved in learning through NF (Koralek et al., 2012, 2013; Harmelech et al., 2013; Megumi et al., 2015).

Since the first application of NF in the 1960’s, this method has been the subject of continuing development in clinical application as well as in the field of neuroscience. In the course of animal sleep research, Barry Sterman, research psychologist at the University of California, observed that a certain brain activity called sensorimotor rhythm (SMR) correlated in cats with the distinct behavior of being motorically idle (Roth et al., 1967). Training of this brain activity by operant conditioning not only changed waking and sleep behavior, but the same cohort of animals turned out in a later experiment to be less susceptible to a seizure-inducing agent (Sterman et al., 1969; Sterman and Friar, 1972). This unexpected finding gave impetus to the first human trials, and opened an ongoing development in clinical application, engineering, and neuroscience (for a recent review see Sitaram et al., 2017).

The potential of NF to alter neural signals (McAdam et al., 1966; Kamiya, 1968) and mental states as well as associated behavior makes it a strong candidate as a new therapeutic tool for the treatment of a wide range of symptoms and disorders, including psychiatric and neurologic conditions. Clinical research mainly focused on the use of NF in conditions such as epilepsy (Egner and Sterman, 2006), ADHD (Monastra et al., 2002; Arns et al., 2009; Hodgson et al., 2012; Sonuga-Barke et al., 2013; Micoulaud-Franchi et al., 2015; Cortese et al., 2016), and PTSD (Peniston and Kulkosky, 1991; Kluetsch et al., 2014; Nicholson et al., 2016, 2017; van der Kolk et al., 2016) and addictions (Scott et al., 2005). Symptoms that are part of the frontal dysfunction syndrome have been shown to benefit from NF, e.g., an impaired impulse control and attention deficits in patients with attention deficit hyperactivity disorder (ADHD) (Arns et al., 2014). Moreover, NF was reported to improve affect regulation in patients with chronic posttraumatic stress disorder (PTSD) (van der Kolk et al., 2016). Concerning the use of NF in patients with traumatic brain injury (TBI), several studies reported positive findings providing different modalities of NF (May et al., 2013; Ali et al., 2020). However, the cited reviews claimed that none of the published protocols have been compared with a sham control-group and robust clinical evidence is still lacking. Most recently, an interesting retrospective study reported significant treatment response using ILF-NF in 59 patients with post-concussion symptoms (Legarda et al., 2022). The NF-group experienced significantly greater improvements concerning headache, memory impairment and brain fog than the TAU-group.

Another reason for the limited generalization of evidence for the efficacy of NF is the heterogeneity of NF devices and the ongoing debate as to which parameters of the electroencephalogram (EEG) should be chosen for feedback. Based on the original experiments by Barry Sterman, operant conditioning of EEG frequency bands was used in order to treat many different symptoms and disorders. The attempt to relate specific conditions to certain patterns in the EEG spectrum by quantitative electroencephalography (qEEG) analysis has not led to conclusive results so far (Rogala et al., 2016). At the same time, knowledge has emerged concerning complex systems and dynamical networks, which initiated a shift in understanding brain function and dysfunction, ultimately influencing the clinical view on psychiatric disorders and on brain injury. For example, specific brain functions could be assigned to the classical Brodmann areas, but proper functioning requires the interconnection between them. The disruption of networks, especially in the three large-scale brain networks [default mode network (DMN), executive control network (ECN), and salience network (SN)] is currently regarded as crucial for a variety of brain dysfunctions (Menon, 2011).

### Infra-low frequency neurofeedback

In recent years the Infra-low Frequency NF-method (ILF-NF) has aroused increasing interest of clinicians and scientists alike. The ILF-NF method originates from classical EEG frequency band training, but also takes into account the above-mentioned model of the brain as a dynamical, self-regulating network (Othmer et al., 2013). Feedback is based on the dynamics of the frequency range from 0.5–40 Hz as well as from signals in the infra-low frequency (ILF) range. There are several indications that link the signal in the ILF range to fluctuations of the DMN activation as detected in fMRI (Buckner et al., 2008; Othmer et al., 2013). The regulation of such a core network, given its engagement with, and influence on, the other core regulatory networks like the SN and the CEN presents an attractive model, since dysregulation of these networks has been implicated in a number of mental disorders (Broyd et al., 2009; Menon, 2011). The ability of ILF-NF to regulate core networks is further supported by a recent fMRI study with participants receiving either ILF-NF or sham-NF (Dobrushina et al., 2020). After one session, increased connectivity was found between key regions of the salience, language, and visual networks.

The need for additional therapeutic approaches in the treatment of patients with a frontal brain syndrome and the promising reports concerning the use of NF in emotional, impulsive, and attention regulation as well as recovery from brain injury prompted us to conduct the present study. The main goal was to investigate if NF supports the recovery from a frontal brain lesion. With regard to the spontaneous brain recovery after an injury and the simultaneous application of several therapies during a neurorehabilitation, it is crucial to investigate the potential NF-effect in a placebo-controlled manner. Concerning the NF-method, we decided to use the above mentioned ILF-NF for the following reasons: During the subacute phase after a moderate to severe frontal brain damage, patients often are not able to participate actively in the training. Contrary to the active operant conditioning of the classical NF, the ILF-NF does not require active participation. Secondly, since the training goal in terms of EEG-parameters after a brain injury has not yet been defined, the use of classical NF in this patient group was questionable. Instead, the regulation of brain networks by the use of ILF-NF was deemed a much more suitable approach.

## Materials and methods

### Trial design

A parallel-group, double-blinded, placebo-controlled trial with balanced randomization was conducted at a specialized clinic for neurorehabilitation in Switzerland. Participants were randomly assigned to one of the intervention groups with an allocation ratio of 1:1.

### Participants

This study (NCT02957695 ClinicalTrials.gov) was conducted from June 2015 to February 2017 at the REHAB Basel, a specialized clinic for neurorehabilitation and paraplegiology in Switzerland, in accordance with the Declaration of Helsinki (World Medical Association, 2013). Following the study protocol that had previously been approved by the local ethics committee (EKNZ 2015-105), the participants’ eligibility was assessed by a trained physician, either the responsible clinician or the study physician. Patients with newly acquired moderate to severe frontal or fronto-temporal brain lesions caused by traumatic or non-traumatic conditions aged 18 years or older were eligible. All participants had to be hospitalized for first neurorehabilitation at REHAB Basel during recruitment and had to have acquired their brain lesion one to six months before study inclusion. The physicians in charge clinically judged if the patient demonstrated a sufficient level of vigilance, adequate cognitive skills and sufficient motor function to understand and to perform the required neuropsychological tests. After receiving verbal and written study information, an approved consent form was signed by the patient in case of study participation.

Patients with a history of previous brain injuries, persistent symptomatic epilepsy or severe epilepsy-typical EEG signs, severe cognitive dysfunctions (e.g., sensory aphasia), neurodegenerative diseases, brain cancer, dementia, schizophrenia, and severe abuse of alcohol or drugs were excluded from study participation.

### Clinical characteristics

Besides sociodemographic characteristics three clinical parameters were used to characterize the participants and to evaluate potentially relevant baseline differences between the two intervention groups. These clinical parameters included frontal lesion load, the intake of cognitively relevant medication and the individual’s functional total and cognitive capability at baseline according to the total Functional Independence Measure (FIM) and the cognition subscore of the FIM (for description see Granger et al., 1986; Dodds et al., 1993; Ottenbacher et al., 1996).

The cognitive dysfunctions were ascertained by comprehensive neuropsychological assessments performed during in-patient neurorehabilitation as part of the clinical routine. Relevant medication was judged as present if the patients were permanently treated with one or several pharmaceuticals with well-known influence on cognitive functions such as antiepileptic drugs, benzodiazepines or antipsychotics.

As defined in the study protocol, all study participants (*n* = 20) were required to have at least one cerebral lesion located in the frontal lobes of the brain. As expected in a clinical setting, the 20 participants showed additional lesions in the temporal, parietal, occipital lobes or in subcortical regions. For a further characterization of the lesion, the routinely performed CT or MRI scans were assessed for each patient. Specifically, the area with the largest circumference of each cerebral lesion was determined. Since our interest was focused on frontal damage, we calculated the proportion of the frontal damage area in relation to the total lesion load for each participant. For the subgroup analysis subjects were classified as having predominantly frontal lesions if their frontal brain lesion load accounted for at least 50% of the total damage area. In ambiguous cases (e.g., shearing injuries or several punctuate lesions) the classification was based on the clinical findings. Thus, in these cases a patient’s brain damage was considered as predominantly frontal, if his or her clinical symptoms were predominantly related to frontal brain functions.

### Randomization

Each participant was assigned to an identification number according to the sequence of entry into study. The randomized allocation procedure to one of the two intervention groups was provided by the NF software Cygnet^®^ respective to the identification number with a predetermined ratio of 1:1 within block sizes of 10.

### Blinding

As the allocation procedure was performed by the manufacturer of the NF device, assignment to group was concealed from the participants, care providers and outcome assessors. Since movements or muscle contractions give rise to easily recognizable EEG patterns, such artifacts recorded from the actual EEG were integrated into the sham-NF intervention. In this way, neither the patients nor the investigators were able to detect group affiliation. After study end the allocation was disclosed by the manufacturer without any knowledge of the measurements or analyses.

### Study intervention

**FIGURE 1:**
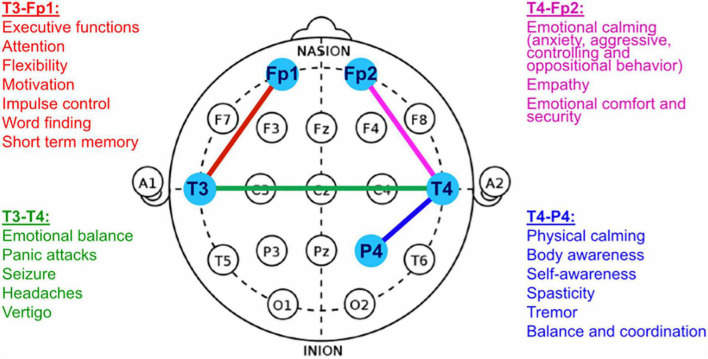
EEG Electrode Positioning for NF-Training (adapted from: Othmer, 2013).

For both study groups (NF and sham-NF, respectively) the setup of the intervention sessions was identical. The NF-method under investigation was the ILF-NF (infra-low frequency neurofeedback) developed clinically and scientifically by Sue and Siegfried Othmer.^1^ Instrumentation consisted of the NeuroAmp II for signal acquisition and Cygnet^®^ software for signal processing and generation of the feedback, and was engineered by B. Wandernoth.^2^ In the training process, the subject’s current brain activity is reflected back in the form of visual, auditory and tactile analogs. Patients were allowed to individually choose their preferred movie for visual and auditory feedback. For the tactile feedback a vibrating teddy bear was provided and held by the patients. EEG electrodes were placed on the head individually according to the ILF-NF Protocol Guide (Othmer, 2013). The optimal reinforcement frequency of the ILF signal was determined individually during the first 1–3 intervention sessions, based either on the patient’s report or on observations of the nurses in charge regarding behavioral alertness. Usually, the reinforcement frequency ranged from 0.1 to 0.5 mHz. As a starting position of the EEG electrodes according to the international 10–20 system either the T3-T4 position or the T4-P4 placement was chosen, depending on the patient’s symptoms. After the individually suitable reinforcement frequency had been determined, 16 sessions of NF-training were conducted. During these 16 sessions, the electrode positions included at least one of the prefrontal positions (T3-Fp1, T4-Fp2, or Fp1-Fp2) next to the basic positions (T4-P4 or T3-T4) (Figure 1).

The NF-system was equipped with a special software providing either real NF or sham-NF according to the patient identification number. In case of sham-NF, the feedback was not calculated from the actually measured EEG, but from a simulated EEG. To ensure blinding, artifacts recorded from the actual EEG were integrated into the sham-NF as well.

### Outcome measures

All outcome assessments were performed twice during the study period, i.e., before and after the intervention phase. The present report comprises the results concerning three cognitive tests *a priori* defined as primary outcomes.

The Frontal Assessment Battery (FAB) is a brief neuropsychological test battery for the assessment of different cognitive functions associated with the frontal brain area (Dubois et al., 2000; Benke et al., 2013). It consists of 6 subscales evaluating conceptualization and abstract reasoning, verbal fluency, motor programming, resistance to conflicting instructions, inhibitory control (Go/NoGo-Paradigm) and environmental autonomy. Each subtest is scored from 0 to 3 points, resulting in a minimum of 0 points for the total FAB score indicating severe frontal dysfunction and a maximum of 18 points indicating no frontal dysfunction at all. A study on patients with various degrees of frontal lobe dysfunction demonstrated good psychometric properties of the FAB with high values for internal consistency (Cronbach’s Alpha = 0.78), interrater reliability (κ = 0.87) and concurrent validity (Bertoux et al., 2013).

The Test of Attentional Performance (TAP) is a neuropsychological test battery comprising 14 distinct aspects of attention. In the current trial, the TAP subtest “alertness” with the two aspects intrinsic alertness and phasic alertness and the TAP subtest “Go/NoGo” were used (For detailed description see Zimmermann and Fimm, 2007). For both tests, good to very good reliability coefficients have been reported (Becker et al., 1996).

### Sample size

Given the clinical setting of the trial at a highly specialized center for neurorehabilitation, the possible sample size was restricted. Moreover, the inclusion criteria were strict and eligible patients had to be able to perform several cognitive tests after recovering from recent moderate to severe brain injuries or illnesses. The goal of the study was to test the null hypothesis that the improvement means of the two intervention groups did not differ significantly. The significance level (alpha) was set at 0.05 and the test two-tailed with a sample size of 10 subjects per group, the study had a power of 78.5% to yield a statistically significant result. This calculation assumed a mean difference of 1.3 for the FAB sum score and a common within-group standard deviation of 1.0. This mean difference was selected as the smallest effect that would be important to detect, in the sense that any smaller effect would not be of clinical or substantive significance. It was also assumed that this effect size was reasonable, in the sense that an effect of this magnitude could be anticipated in this field of research (Uchida and Kawashima, 2008; Chibbaro et al., 2012). Due to the argumentation given above on the study set up and on the assumptions for the calculations, the sample size considered was determined to be 10 subjects per group. For the power calculation, Power and Precision, release 4.1, was used.

### Statistical analysis

Patients’ characteristics were summarized as means (±SD) or medians (±IQR) for continuous variables and as counts and proportions for categorical variables. To evaluate the comparability of the two study groups at baseline, the most relevant sociodemographic and clinical characteristics as well as the level of cognitive impairment (FIM cognition subscore) were determined.

The primary outcome was the improvement of participants in several cognitive functions specific for the frontal lobe. Data distribution was checked for normality. Differences between the two intervention groups regarding the mean change of the measured cognitive outcomes before and after the intervention phase were evaluated using bivariate Generalized Linear Models (GLMs) in a first step and multivariable GLMs in a second step. Multivariable models included (i) treatment group and relevant medication or (ii) treatment group, age, and education. The possibility of an interaction between treatment group and examination time points was examined. This interaction was not significant for the presented outcomes. All analyses were performed both for the whole study sample (*n* = 20) and for the subgroup of patients with mainly frontal brain lesions (*n* = 9). The subgroup analysis for individuals with mainly frontal brain lesion was exploratory because the procedure was not described *a priori* in the study protocol.

In addition, effect sizes were calculated to evaluate the clinical significance of the differences. Klauer (2001) suggested calculating the effect size of pre-post measurements using Hedges g and subtracting the two effect sizes from each other (Klauer, 2001). For the interpretation of the calculated effect sizes the classification according to Cohen (1988) was applied, with a value or r of | 0.2| representing a “small” effect, | 0.5| representing a “medium” effect and | 0.8| representing a “large” effect (Cohen, 1988).

## Results

### Participants

**FIGURE 2:**
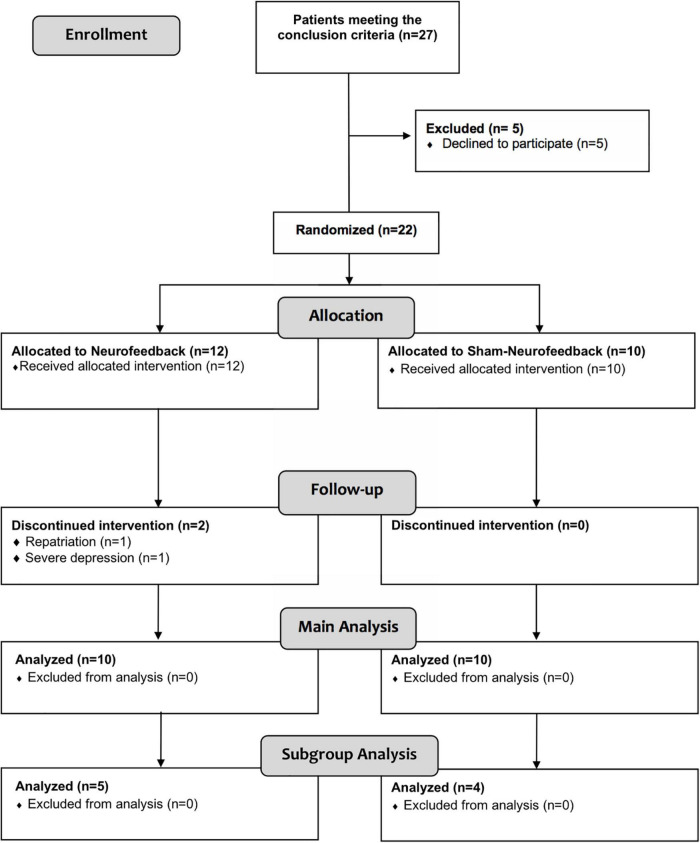
Flowchart showing the number of participants and drop-outs during the course of the trial.

In total, 22 patients were included in the trial. Two patients dropped out after having started with the intervention sessions. Both of them had originally been randomized to receive sham-NF. One patient was repatriated to the United States, the other one turned out to suffer from severe depression, preventing further study participation. Thus, 20 patients (10 NF, 10 sham-NF) completed the study protocol and were analyzed (see Figure 2). The subgroup analysis of patients with predominantly frontal brain lesions comprised 9 individuals (5 NFB, 4 sham-NF).

### Total study sample

#### Demographic and clinical characteristics

Before applying the NF-intervention, we assessed all patients concerning sex, age, education, etiology and location of brain lesions, FIM score and the level of neurologic and neuropsychologic deficits. Most of the participants were male with a proportion of 80% men in each of the intervention groups. The mean age of the total sample was 40.3 years (SD 16.9) with the NF group being slightly older than the sham group (43.5 ± 16.9 vs. 37 ± 17.1 years, *p* = 0.472). The mean years of education in the NF group were 12.9 ± 2.3 versus 12 ± 2.6 in the sham group (*p* = 0.267). Relevant medication was present in two patients from NF group versus four patients of the sham group (*p* = 0.629). The etiology of the brain lesions was equally distributed in the sham group (50% each for traumatic and non-traumatic etiology) but not in the NF group with 90% traumatic lesions (*p* = 0.472). The mean total FIM score at baseline was 86.1 ± 19.5 in the NF group and 78.9 ± 25.6 in the sham group (*p* = 0.545). The FIM cognition subscore was 21.5 ± 6.5 in the NF group versus 21.9 ± 4.9 in the sham group (*p* = 0.970). In summary, there were no statistically significant differences between the two intervention groups regarding any of these clinical and demographic characteristics.

The results of the clinical presentation of all patients are depicted in Table 1. Summarized are the neurologic and neurocognitive deficits, the total score and the cognitive subscore of the FIM as well as the proportion of the frontal damage in proportion of the total lesion load. The participants that showed a proportion > 50% were selected for the subgroup analysis and marked in bold. Concerning all patients, their deficits comprised a wide variety of somatic and cognitive impairments including hemiparesis, hemineglect, aphasia, and deficits in spatial cognition or comprehension of complex issues. These can be ascribed to non-frontal injuries as well as to the typical frontal brain deficits concerning attention, memory, executive function, behavior and emotion control. In the subgroup of patients classified as having predominantly frontal brain lesions (*n* = 9), the proportion of frontal lesion load in relation to total damage ranged from 67 to 100%. In these patients, the resulting neurological deficits mainly comprised attention deficits and impairments in executive functions and behavioral control.

**TABLE 1:** Clinical presentation of all study participants (*n* = 20).

ID	Main neurological deficits (somatic and cognitive)	FIM (max. 126)	FIM Cognition (max. 35)	Proportion of frontal brain damage in relation to total lesion load (%)
1	Right-sided hemiparesis; aphasia; severe deficits in executive function (flexibility, impulse control, and planning); moderate to severe deficits in learning, memory and attention; anosognosia	68	21	32
2	Moderate to severe deficits in spatial perception, executive function, attention and memory, behavior control; left-sided neglect	74	25	**68**
3	Moderate to severe deficits in attention, memory, executive functions, and comprehension of complex issues; anosognosia	106	26	38
4	Tetraparesis left > right; motor aphasia; left-sided neglect; severe brain dysfunction with deficits in attention and memory, flexibility, idea production, cognitive control, and resilience	77	27	**87**
5	Minor to moderate deficits in many aspects of brain function, e.g., attention and concentration, motivation, empathy, spatial recognition, and executive functions	117	27	15
6	Severe deficits mainly in attention and memory functions, executive functions, behavior control, impulse control, and orientation ability	63	8	**67**
7	Moderate to severe deficits mainly in attention and memory functions, executive functions and impulse control	95	18	**100**
8	Left-sided sensorimotor hemiparesis and hemineglect; reduced vigilance; moderate to severe deficits in attention and flexibility and comprehension	70	19	13
9	Left-sided hemineglect; minor to moderate deficits in attention, memory and executive functions	106	26	Not definable (shearing injuries)
10	Left-sided sensorimotor hemiparesis; hemineglect and hemianopsy; moderate deficits in attention, memory and executive functions and visual-motor skills	39	14	36
11	Left-sided sensorimotor hemiparesis and hemineglect; severe deficits in attention, memory, executive functions and behavior control; personality change; anosognosia	62	18	**72**
12	Left-sided sensorimotor hemiparesis and hemineglect; severe deficits in visual-motor skills; moderate deficits in attention and executive functions	53	20	43
13	Right-sided sensorimotor hemiplegia; global aphasia; severe deficits in language-associated skills; minor deficits in attention and executive functions	84	22	7
14	Minor to moderate deficits in attention, memory and behavior control	64	22	**80**
15	Moderate to severe deficits in attention, memory, executive functions, behavior control and emotion control; severe anosognosia	80	17	**100**
16	Moderate to severe deficits in memory; moderate deficits in executive functions; mild attention deficits	99	25	30
17	Mild deficits in attention; reduced resilience	118	29	Not definable (fronto-parietal punctate hemorrhages)
18	Mild to moderate deficits in attention and executive functions; moderate deficit in visual-motor skills; anosognosia	107	29	28
19	Severe deficits in memory; moderate to severe deficits in executive functions; moderate deficits in attention and mild deficits in behavior control	102	26	**100**
20	Tetrapyramidal syndrome; moderate deficits in attention, memory and executive functions, impulse control, emotion control and behavior control	66	15	**Not definable (punctate hemorrhages)**

#### Cognitive outcomes

As presented in Table 2, the cognitive measures at baseline were similar in both study groups. A statistically significant baseline difference was only present for the TAP phasic alertness parameter, with the NF group starting from a lower average level than the sham group (−0.01 ± 0.13 vs. 0.14 ± 0.16, *p* = 0.034).

**TABLE 2:** Baseline values and unadjusted GLM analysis of the mean cognitive improvement.

Outcome measure	Sham-Neurofeedback (*n* = 10)		Neurofeedback (*n* = 10)		Mean difference between groups at study end (95% CI)	*P-value*	Effect size d_*corr*_ sensu Klauer
	Baseline, mean ± SD	Study End, mean ± SD	Baseline, mean ± SD	Study End, mean ± SD			
FAB, Total score	13.7 ± 4.2	15.6 ± 2.7	12.0 ± 4.0	16.5 ± 1.9	2.6 (−0.2 to 5.4)	0.068	0.80
TAP Intrinsic Alertness, reaction time (ms)	461.0 ± 217.9	411.1 ± 257.4	373.5 ± 134.3	336.0 ± 94.2	12.4 (−128.2 to 153.0)	0.855	0.10
TAP Phasic Alertness, parameter	0.14 ± 0.16	0.10 ± 0.13	−0.01 ± 0.13	0.04 ± 0.07	0.09 (−0.01 to 0.20)	0.079	0.45
TAP Go/NoGo, (no. of mistakes)	1.7 ± 1.6	0.8 ± 1.0	1.4 ± 1.5	0.8 ± 1.1	0.2 (−1.5 to 2.0)	0.781	0.19

Total study sample (*n* = 20). FAB, Frontal Assessment Battery, GLM, Generalized Linear Model, SD, Standard Deviation, CI, Confidence Interval, TAP, Test of Attentional Performance.

**FIGURE 3:**
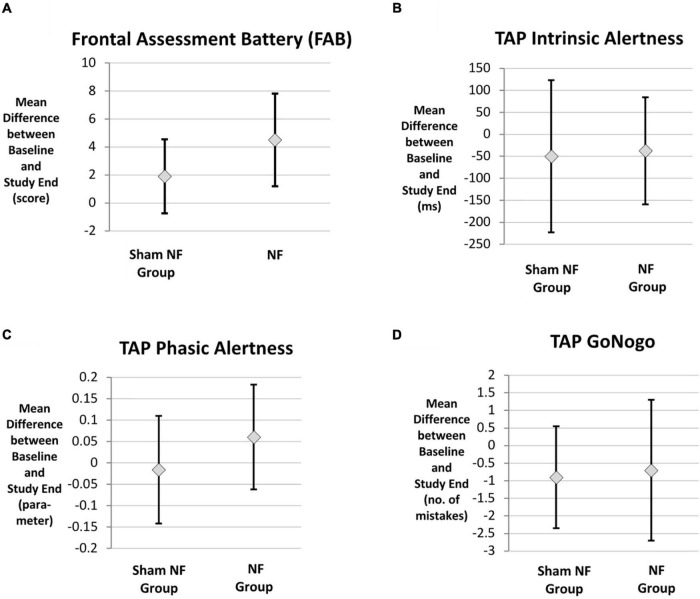
Cognitive improvement in both intervention groups. **(A)** Frontal Assessment Battery (FAB), **(B)** TAP intrinsic alertness, **(C)** TAP phasic alertness, **(D)** TAP Go/NoGo, *n* = 20. TAP, Task of Attentional Performance.

During the course of the trial, the participants’ average cognitive performance improved in both intervention groups (Table 2). With respect to all cognitive tasks, the mean improvement during the trial did not differ significantly between the two intervention groups based on both the unadjusted GLM analysis (all *p*-values > 0.05, Table 2) and the GLM adjusted for relevant medication (Table 3). The graphical representation of the cognitive improvement during the trial in both intervention groups is displayed in Figures 3A–D.

**TABLE 3:** Adjusted GLM analysis of the mean cognitive improvement adjusted for relevant medication.

Outcome measure	Adjusted mean difference between groups at study end (95% CI)	*P-value*
FAB, Total score	2.6 (−0.4 to 5.5)	0.086
TAP Intrinsic Alertness, reaction time (ms)	−24.7 (−145.6 to 96.2)	0.672
TAP Phasic Alertness, parameter	0.08 (−0.03 to 0.18)	0.139
TAP Go/NoGo (no. of mistakes)	0.1 (−1.7 to 1.9)	0.912

Total study sample (*n* = 20). GLM, Generalized Linear Model, CI, Confidence Interval, FAB, Frontal Assessment Battery, TAP, Test of Attentional Performance.

### Subgroup analysis: Patients with mainly frontal brain lesions

#### Demographic and clinical characteristics

The subgroup analysis restricted to 9 patients with mainly frontal brain lesions consisted exclusively of men. Five of these individuals were randomized to the NF group and 4 to the sham group. There were no statistically significant differences at baseline between the two intervention groups regarding age, education years, relevant medication and etiology of the brain lesion (data not shown). The FIM Cognition Subscore at baseline also did not differ significantly but the NF group started with a lower mean value than the sham group (17.6 ± 6.9 vs. 22.0 ± 4.7, *p* = 0.219).

In accordance with the FIM Cognition Subscore there was also a baseline difference regarding the FAB total score between both intervention groups. The NF group started with a lower mean FAB score than the sham group (9.2 ± 1.8 vs. 15.3 ± 3.1, *p* = 0.025) (Table 4).

**TABLE 4:** Subgroup Analysis: Baseline values and unadjusted GLM of the mean cognitive improvement in patients with predominantly frontal brain lesions (*n* = 9).

Outcome measure	Sham-Neurofeedback (*n* = 4)		Neurofeedback (*n* = 5)		Mean difference between groups at study end (95% CI)	*P-value*	Effect size d_*corr*_ sensu Klauer
	Baseline, mean ± SD	Study End, mean ± SD	Baseline, mean ± SD	Study End, mean ± SD			
FAB, Total score	15.3 ± 3.1	15.3 ± 3.4	9.2 ± 1.8	16.0 ± 2.4	6.8 (3.7 to 9.9)	**0.001**	**2.74**
TAP Intrinsic Alertness, reaction time (ms)	452.5 ± 162.8	384.2 ± 221.9	463.2 ± 139.8	355.0 ± 102.4	40.0 (−219.8 to 139.9)	0.616	−0.25
TAP Phasic Alertness, parameter	0.16 ± 0.18	0.12 ± 0.14	−0.07 ± 0.11	0.06 ± 0.06	0.19 (0.09 to 0.28)	**0.002**	**1.01**
TAP Go/NoGo (no. of mistakes)	1.3 ± 1.9	0.8 ± 1.0	2.2 ± 1.6	0.5 ± 0.6	−1.5 (−4.4 to 1.4)	0.254	−0.90

GLM, Generalized Linear Model, CI, Confidence Interval, FAB, Frontal Assessment Battery, TAP, Test of Attentional Performance. Bold values are significant result.

#### Cognitive outcomes

**FIGURE 4:**
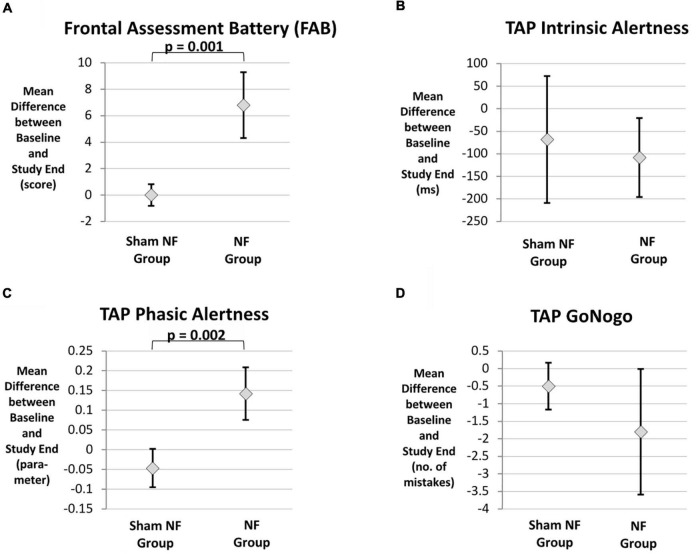
**(A–D)** Cognitive improvement in the subgroup of patients with mainly frontal brain lesions (FAB), TAP intrinsic alertness, TAP phasic alertness, TAP Go/Nogo, *n* = 9.

As displayed in Table 4 (unadjusted GLM analysis), the between-group comparisons resulted in statistically significant differences regarding the improvement in the FAB total score (*p* = 0.001) and the TAP phasic alertness parameter (*p* = 0.002) (Figures 4A,C). The effect sizes of these two between-group differences were large (*d* = 2.74 and *d* = 1.01, respectively).

Regarding TAP intrinsic alertness, only the adjusted GLM indicated a significant difference in favor of the NF group (*p* = 0.047) (Tables 4, 5 and Figure 4B). There were no statistically significant differences between the two intervention groups concerning the improvement in inhibitory control as measured by the number of mistakes in the TAP Go/NoGo task (Tables 4, 5 and Figure 4D).

**TABLE 5:** Subgroup Analysis: Adjusted GLM of the mean cognitive improvement adjusted for relevant medication in patients with predominantly frontal brain lesions (*n* = 9).

Outcome measure	Adjusted mean difference (95% CI) between groups at study end	*P-value*
FAB, Total score	6.3 (3.0 to 9.7)	**0.004**
TAP Intrinsic Alertness, reaction time (ms)	−99.5 (−197.0 to −2.0)	**0.047**
TAP Phasic Alertness (parameter)	0.18 (0.07 to 0.29)	**0.007**
TAP Go/NoGo (no. of mistakes)	−1.4 (−4.8 to 2.1)	0.353

GLM, Generalized Linear Model, CI, Confidence Interval, FAB, Frontal Assessment Battery, TAP, Test of Attentional Performance. Bold values are significant result.

### Sensitivity analysis: Adjustment for age and education

After alternatively adjusting the GLM for age and education instead of relevant medication the effect estimates did not change substantially (data not shown).

## Discussion

For the total study sample (*n* = 20), the cognitive improvement after the intervention phase did not differ significantly between the NF group and the sham intervention group regarding all cognitive outcomes under investigation. Concerning the FAB and the phasic alertness, larger improvements were observed in the NF group, which did not reach significance (*p* = 0.068, *p* = 0.079, respectively). When analyzed in the subgroup of patients with predominantly frontal lesions (*n* = 9), the NF group showed a significantly larger improvement measured by these two tests (*p* = 0.001, *p* = 0.002, respectively). The observed effects in terms of the FAB and attention are in line with reported effects of NF concerning attention and executive functions within children or adolescents with ADHD (Riesco-Matías et al., 2019; Van Doren et al., 2019) and emotional regulation in patients with PTSD (van der Kolk et al., 2016). However, the results of the subgroup analysis *per se* have the major limitation of the small number of participants. Interestingly, the significantly different outcomes were obtained by the same two tests which showed already a trend in the whole group study. Taken together, our results represent an encouraging outcome after 20 sessions of NF, making NF a promising candidate supporting the recovery from a frontal brain injury.

During the course of the trial, the NF intervention was well tolerated. No significant side-effects were observed and there was no dropout because of the intervention itself. Interestingly, the patients with their well-known problems in self-regulation did not show any compliance problems in participating and completing the NF-sessions.

## Strength and limitations

To the best of our knowledge, the present study was the first double-blind, sham-controlled clinical trial evaluating the use of ILF-NF training among patients with recently acquired moderate or severe brain damage. Furthermore, the analyses comprised information about a variety of potentially relevant sociodemographic and clinical characteristics of the participants like years of education, medication, or baseline FIM scores. These characteristics have been considered in the adjusted GLM-analysis.

There are major limitations to be mentioned in this relatively small study. Although the sample size calculation showed a power of 78.5% to yield a statistically significant result with a sample size of 10 participants for each of the two groups, this sample size has to be regarded as small, considering the high heterogeneity of the participants regarding localization and extent of the brain lesions. In the present study, all of the participants showed frontal brain lesions, but several of them had relevant additional lesions that resulted in sensorimotor hemiparesis, neglect, hemianopsia or impairment in parietal functions like perception or cognitive functions like calculation or reading. The subgroup analysis was conducted in order to address the potential bias of non-frontal additional lesions. In this subgroup, a significant difference in the outcome between the two groups was measured by the FAB, the TAP phasic and intrinsic alertness. However, corresponding to the resulting small sample size, these effect estimates showed wide confidence intervals indicating a considerable level of uncertainty about the true underlying association. Further studies with larger groups and less heterogeneity concerning the localization and extent of brain lesions are needed.

Additionally, for the subgroup of patients with mainly frontal lesions a ceiling effect regarding the FAB total score cannot be excluded. As presented in Table 4, the patients randomized to sham-NF started with a significantly higher mean FAB score than the patients in the NF group. Therefore, the participants receiving NF may have had a larger potential for cognitive improvement during the time period of the trial.

## Conclusion and future perspectives

No clear-cut conclusion can be drawn from the presented study. However, the encouraging result from the subgroup analysis as well as the simple and well tolerated application suggest that NF could be a promising candidate to support the recovery from frontal brain injury during neurorehabilitation. Finding alternative treatments for the frontal brain dysfunction is particularly important in light of the limitations of existing treatments. We therefore suggest that NF deserves further studies to substantiate his effectiveness and its underlying mechanisms.

Concerning mechanisms of brain recovery, principles of neuronal plasticity have been well described, and likely serve as the underlying mechanism of NF effects as well (see also section “Introduction”). In contrast to the knowledge base at the molecular and the network level, still little is known about the mechanisms at the regional level in the brain, especially concerning the prioritization of brain recovery when several brain areas or brain functions are impaired (Rossini et al., 2007; Hara, 2015). In the present study, the training sites were selected according to the clinical presentation, with focus on frontal deficits. Impairments like hemiparesis were not specifically trained, although basic positions like T3-T4 may have an impact to such impairments as well. A better understanding of the dynamics in brain recovery would not only benefit the timely organization of rehabilitation in general but also would guide the therapeutic approach of NF in terms of the training sequence of regions and training time in patients with brain injuries.

## Data availability statement

The raw data supporting the conclusions of this article will be made available by the authors, without undue reservation.

## Ethics statement

The studies involving human participants were reviewed and approved by the Ethikkommission Nordwest- und Zentralschweiz EKNZ. The patients/participants provided their written informed consent to participate in this study.

## Author contributions

CA: initiation of the study, substantial contribution to the conception, the acquisition, and analysis and interpretation of data for the work as well as writing the most part of the article. KH: drafting the work and revising it critically for important intellectual content and for ethical considerations. CS: substantial contribution to the analysis of data and writing of some parts of methods and results. MM: substantial contribution to the conception and mainly acquisition of the data. YS: substantial contribution to the acquisition and analysis of the data. MH-G: giving substantial inputs to the interpretation of data and providing approval for publication of the content. All authors contributed to the article and approved the submitted version.

## Footnotes

1
www.eeginfo.com

2
www.BEE-medic.de
